# Occupational therapy and eating disorders: an integrative literature review

**DOI:** 10.1186/2050-2974-3-S1-P8

**Published:** 2015-11-23

**Authors:** Elysa Roberts, William Wolfenden

**Affiliations:** 1 University of Newcastle, Newcastle, Australia; 2 Bondi Junction Community Mental Health Centre, Sydney, Australia

## Introduction

Literature pertaining to the role of occupational therapy in recovery from eating disorders spans 40 years; however, has not been systematically synthesized.

## Methods

To develop a comprehensive understanding of reported roles of occupational therapy in recovery from eating disorders and inform intervention and research, we conducted an integrative literature review. Searches were conducted in ten online databases and six occupational therapy journals. Theoretical or empirical publications in peer-reviewed journals in English explicitly describing occupational therapy intervention or role of the occupational therapist for eating disorders were included. Thematic analysis was conducted across selected articles and research articles critically reviewed.

## Results

Thirty-two pieces of evidence published in English from 1974 through 2014 were included in this review; twenty-four were theoretical and eight were qualitative, descriptive or quasi-experimental studies. Analysis revealed the domain and process of occupational therapy with persons with eating disorders. Results are represented as a conceptual model illustrating a variation of the American Occupational Therapy Association Practice Framework. Appraisal of the studies suggests a need and opportunity to document and disseminate the evidence-base for this practice area.

## Conclusion and significance

Findings of this integrative literature review inform research, practice and clinical training of occupational therapists in the specialty area of eating disorder recovery.

